# Analysis of factors associated with the use of Korean medicine after spinal surgery using a nationwide database in Korea

**DOI:** 10.1038/s41598-023-47454-5

**Published:** 2023-11-17

**Authors:** Doori Kim, Yoon Jae Lee, Bo-Hyoung Jang, Jeong-Su Park, Sunju Park, Christopher R. D’Adamo, Yong Cheol Shin, Seong-Gyu Ko

**Affiliations:** 1 Department of Preventive Medicine, College of Korean Medicine, Kyung Hee University, 26 Kyungheedae-ro, Dongdaemun-gu, Seoul, 02447 Republic of Korea; 2 Jaseng Spine and Joint Research Institute, Jaseng Medical Foundation, 540 Gangnam-daero, Gangnam-gu, Seoul, 06110 Republic of Korea; 3 Department of Preventive Medicine, College of Korean Medicine, Semyung University, 65, Semyeong-ro, Jecheon-si, Chungcheongbuk-do Republic of Korea; 4 Department of Preventive Medicine, College of Korean Medicine, Daejeon University, 62, Daehak-ro, Dong-gu, Daejeon, 34520 Republic of Korea; 5 Department of Family and Community Medicine, University of Maryland School of Medicine, 655 West Baltimore Street, Baltimore, MD 21201 USA

**Keywords:** Health care, Pain

## Abstract

Many patients in Korea use Korean Medicine (KM) after spine surgery, but related research is lacking. Therefore, this retrospective cohort study aimed to analyze factors affecting the use and costs of KM using nationally representative data from the National Health Insurance Service-National Sample Cohort, South Korea. Patients who underwent spinal surgery for spinal diseases from 2011 to 2014 were followed up for 5 years, and their medical care was described. The association between patient and spinal surgery characteristics and the use of KM was analyzed. A two-part model was used to analyze factors affecting the use of KM in patients undergoing spinal surgery. Of 11,802 patients who underwent spinal surgery, 11,367 who met the inclusion criteria were included. Overall, 55.5% were female, 32.3% were aged ≥ 70 years, and 50.2% received KM treatment during the follow-up period. Open discectomy was the most common surgical procedure performed (58.6%), and 40.2% of surgeries were performed because of lumbar disc disorder. Female sex, older age, high Charlson Comorbidity Index score, and use of KM before surgery were associated with increased KM use and expenditure after surgery. In conclusion, patient characteristics, rather than surgical characteristics, appeared to be more strongly associated with the use of KM after surgery, particularly prior experience with KM use. This study is significant in that it analyzed the entire spine surgery to provide a comprehensive view of the use of KM after spine surgery and analyzed the impact of various factors related patients and surgical characteristics on KM use. The results of this study may be useful to patients with spinal diseases, clinicians, and policymakers.

## Introduction

Back pain is one of the most common causes of chronic pain in adults and is considered an important global health problem owing to its high prevalence and socioeconomic cost^1^. Back pain is also a major cause of disability. The number of years lived with disability in 2015 was 60.1 million, an increase of approximately 54% compared to that in 2005^2^. In addition, the lifetime prevalence of back pain is as high as 66%^3^, although it varies from study to study. Back pain is associated with decreased productivity, increased medical expenses, and long-term opioid use^2,4–6^.

Non-specific back pain is common and associated with spinal diseases, such as intervertebral disc disorders and spinal stenosis, in many cases. Spinal surgery is one of the primary treatment methods for spinal diseases. In many countries, spinal surgery and surgical costs have increased in recent decades^7–9^. In the United States, spinal fusion surgeries increased by 62.3% between 2004 and 2015, and the cost of spinal surgery in 2015 was $10 billion^8^. According to the 2020 Main Surgery Statistics Yearbook of the National Health Insurance Service, the number of general spine surgeries in Korea was 188,394, the second highest after cataract surgery, and the cost of surgery was the highest at 918.2 billion South Korean won (KRW)^10^.

As several patients experience pain following spinal surgery^11^, pain management after spinal surgery is very important^12^. Many patients manage pain by taking opioids, such as morphine, after spinal surgery^13,14^. According to a study, more than half of those who used opioids before surgery took opioids 12 months after surgery^13^, and another study showed that more than 25% of patients took opioids 1 year after spinal surgery^14^. Medications such as non-steroidal anti-inflammatory drugs and paracetamol are often administered when opioids are being avoided^15^. Additionally, patients may undergo reoperation or readmission for various reasons^16–18^. The 10-year spinal reoperation rate in Korea was 13.2%; patients in their 60 s had the highest risk^19^.

South Korea has a dichotomized healthcare system with both western and Korean medicine (KM); the latter is one of the traditional medicine systems used in East Asia along with traditional Chinese medicine. In particular, KM modalities, such as acupuncture, herbal medicine, and Chuna treatment are widely used as conservative treatments for spinal diseases^20–23^. According to the 2020 health insurance statistics, back pain is the most frequent indication for the use of KM^23^.

In particular, many patients receive KM treatment after spinal surgery^24,25^. However, little is known about the characteristics of patients using KM after spine surgery and the cost of using KM. Understanding this can be helpful to KM clinicians who treat patients in real world and policymakers who establish policies related to coverage and accessibility of KM treatment.

Therefore, this study aimed to characterize the medical care after spinal surgery in Korea and to analyze factors associated with the use of KM after spinal surgery based on data from the National Health Insurance Service–National Sample Cohort (NHIS-NSC), South Korea.

## Results

### Participants

**Figure 1:**
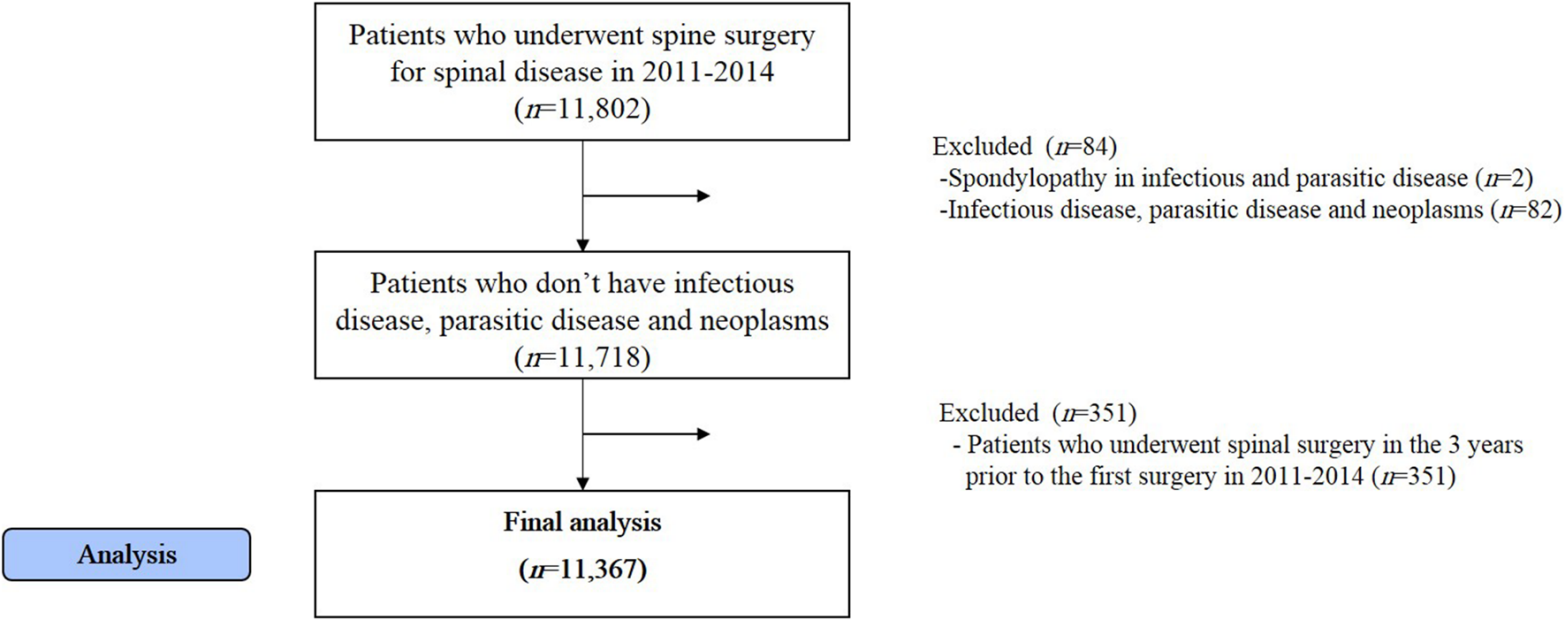
Participants of the study. A total of 11,802 patients underwent spinal surgery for spinal diseases from 2011 to 2014, 11,367 patients were included in the analysis.

A total of 11,802 patients underwent spinal surgery for spinal diseases from 2011 to 2014. Patients with spondylopathy (n = 2), other infections, parasitic diseases, and malignant or benign neoplasm (n = 82) were excluded. Patients with a history of spinal surgery within 3 years prior to the first surgery between 2011 and 2014 (n = 351) were also excluded. Finally, 11,367 patients were included in the analysis (Fig. 1).

### Patient characteristics associated with KM use

The characteristics of the participants are presented in Table 1. Among 11,367 patients, 6311 (55.5%) were female, and the average age was 59.77 ± 15.93 years. There was a monotonic increase in the frequency of spinal surgery across age categories, with the highest frequency observed in the > 70 years group (32.3%). In addition, 4963 patients (43.7%) had used KM in the year prior to surgery.

**Table 1:** Basic characteristics of study participants.

	Total	KM use	Non-KM use	*p*-value
	N (%)	N (%)	N (%)	
Total	11,367	5711	5656	
Sex				
Male	5056 (44.5)	2244 (39.3)	2812 (49.7)	< .0001
Female	6311 (55.5)	3467 (60.7)	2844 (50.3)	
Age				
Mean ± SD (years)	59.77 ± 15.93	61.09 ± 14.75	58.43 ± 16.93	< .0001
0–19	144 (1.3)	35 (0.6)	109 (1.9)	< .0001
20–29	375 (3.3)	143 (2.5)	232 (4.1)	
30–39	959 (8.4)	396 (6.9)	563 (10.0)	
40–49	1448 (12.7)	653 (11.4)	795 (14.1)	
50–59	2360 (20.8)	1145 (20.0)	1215 (21.5)	
60–69	2414 (21.2)	1370 (24.0)	1044 (18.5)	
≥ 70	3667 (32.3)	1969 (34.5)	1698 (30.0)	
Income				
Level 1 (low)	2501 (22.0)	1265 (22.2)	1236 (21.9)	0.1826
Level 2	2743 (24.1)	1329 (23.3)	1414 (25.0)	
Level 3	2682 (23.6)	1360 (23.8)	1322 (23.4)	
Level 4	3368 (29.6)	1721 (30.1)	1647 (29.1)	
Region				
Seoul	1752 (15.4)	825 (14.4)	927 (16.4)	< .0001
Capital area	3222 (28.3)	1499 (26.2)	1723 (30.5)	
Metropolitan city	2052 (18.1)	1102 (19.3)	950 (16.8)	
Others	4341 (38.2)	2285 (40.0)	2056 (36.4)	
CCI^3^				
0	2264 (19.9)	940 (16.5)	1324 (23.4)	< .0001
1	2748 (24.2)	1329 (23.3)	1419 (25.1)	
2	2330 (20.5)	1193 (20.9)	1137 (20.1)	
3	1562 (13.7)	878 (15.4)	684 (12.1)	
≥ 4	2463 (21.7)	1371 (24.0)	1092 (19.3)	
Use of KM before surgery				
None	6404 (56.3)	2485 (43.5)	3919 (69.3)	< .0001
KM use	4963 (43.7)	3226 (56.5)	1737 (30.7)	
Time to the use of KM after surgery				
Mean ± SD (days)	–	522.05 ± 490.23	–	
Median (Q1, Q3)	–	356 (109, 840)	–	
Within 1 year	–	2890 (50.6)	–	
Between 1 and 3 years	–	1897 (33.2)	–	
Between 3 and 5 years	–	924 (16.2)	–	
Time to medical service use after surgery				
Mean ± SD (days)	38.33 ± 148.24	31.05 ± 112.05	46.01 ± 178.32	< .0001
Median (Q1, Q3)	9 (4, 19)	8 (4, 19)	9 (5, 20)	–
Within 1 year	10,876 (97.7)	5617 (98.4)	5259 (93.0)	< .0001
Between 1 and 3 years	171 (1.5)	74 (1.3)	97 (1.7)	
Between 3 and 5 years	80 (0.7)	20 (0.4)	60 (1.1)	

*CCI* Charlson Comorbidity Index, *KM* Korean medicine.

A total of 5711 patients received KM treatment during the 5-year follow-up period, accounting for 50.2% of all patients who underwent surgery. In the KM use group, the proportions of females (60.7%), patients aged > 60 years (58.5%), patients residing in metropolitan cities (19.3%), others (40%), and patients with CCI scores > 3 (39.4%) were high. In the KM use group, the average time from discharge to the first use of KM was 522.05 ± 490.23 days, and the median number was 356 days.

### Characteristics of index spine surgery according to the use of KM

The characteristics of the index spinal surgeries are presented in Table 2. Open discectomy was the most common (6661 patients, 58.6%), followed by fusion (3025 patients, 26.6%), percutaneous vertebroplasty (2385 patients, 21.0%), and laminectomy (2171 patients, 19.1%). Regarding disease type, 40.2% of the surgeries were due to lumbar disc disorder, and by disease sites, 80% were performed in the lumbar spine (l-spine) area. The average length of hospitalization and surgical costs were 14.20 ± 11.11 days and 2537.73 ± 1997.66 US dollars, respectively. In the KM use group, more patients underwent surgery for stenosis than in the non-KM use group, and by surgical site, more patients underwent surgery for the lumbar spine (p < 0.05). In addition, average length of hospitalization and hospitalization costs were quite long (14.57 ± 11.13 days) and high (2555.24 ± 1920.01 US dollars), respectively, in the KM use group.

**Table 2:** Characteristics of index spine surgery.

	Total	KM use	Non-KM use	
	N (%)	N (%)	N (%)	*p*-value
Total	11,367	5711	5656	
Types of surgery				
Fusion	3025 (26.6)	1489 (26.1)	1536 (27.2)	0.4922
Open discectomy	6661 (58.6)	3321 (58.2)	3340 (59.1)	0.8011
PELD	134 (1.2)	69 (1.2)	65 (1.1)	0.6862
Nucleolysis	21 (0.2)	9 (0.2)	12 (0.2)	0.5267
Pt_plasty	2385 (21.0)	1219 (21.3)	1166 (20.6)	0.1371
Laminectomy	2171 (19.1)	956 (16.7)	1215 (21.5)	< .0001
C_plasty	90 (0.8)	44 (0.8)	46 (0.8)	0.8685
Corpectomy	46 (0.4)	26 (0.5)	20 (0.4)	0.3579
Reduction	37 (0.3)	22 (0.4)	15 (0.3)	0.2371
Others	39 (0.3)	17 (0.3)	22 (0.4)	0.4403
Complexity of surgery				
Percutaneous	2492 (21.9)	1281 (22.4)	1211 (21.4)	0.1764
Open (simple)	5850 (51.5)	2886 (50.5)	2964 (52.4)	
Open (w. instrument)	3025 (26.6)	1489 (26.1)	1536 (27.2)	
Types of disease				
Lumbar disc disorders	4573 (40.2)	2241 (39.2)	2332 (41.2)	< .0001
Fracture	2471 (21.7)	1169 (20.5)	1302 (23.0)	
Stenosis	2292 (20.2)	1306 (22.9)	986 (17.4)	
Deforming dorsopathies	884 (7.8)	460 (8.1)	424 (7.5)	
Cervical disc disorders	790 (6.9)	364 (6.4)	426 (7.5)	
Spondylosis	92 (0.8)	54 (0.9)	38 (0.7)	
Inflammatory spondylopathies	45 (0.4)	15 (0.3)	30 (0.5)	
Dorsalgia	40 (0.4)	17 (0.3)	23 (0.4)	
Dorsopathy	29 (0.3)	15 (0.3)	14 (0.2)	
Other spondylopathies	151 (1.3)	70 (1.2)	81 (1.4)	
Disease sites				
Cervical spine	1060 (9.3)	475 (8.3)	585 (10.3)	0.0016
Thoracic spine	1043 (9.2)	514 (9.0)	529 (9.4)	
Lumbar spine	9099 (80.0)	4642 (81.3)	4457 (78.8)	
Others	165 (1.5)	80 (1.4)	85 (1.5)	
Type of institutions				
Clinic	305 (2.7)	156 (2.7)	149 (2.6)	0.0002
Hospital	6666 (58.6)	3457 (60.5)	3209 (56.7)	
General hospital	2883 (25.4)	1356 (23.7)	1527 (27.0)	
Tertiary hospital	1513 (13.3)	742 (13.0)	771 (13.6)	
Length of stay (days)				
Mean ± SD	14.20 ± 11.11	14.57 ± 11.13	13.83 ± 11.09	0.0004
Median (Q1, Q3)	12 (8,18)	12 (8,18)	11 (7, 17)	
Q1	2788 (24.5)	1311 (23.0)	1477 (26.1)	< .0001
Q2	2701 (23.8)	1311 (23.0)	1390 (24.6)	
Q3	2993 (26.3)	1564 (27.4)	1429 (25.3)	
Q4	2885 (25.4)	1525 (26.7)	1360 (24.0)	
Costs for surgery (US dollars)				
Mean ± SD	2537.73 ± 1997.66	2555.24 ± 1920.01	2520.05 ± 2073.15	0.3479
Median (Q1, Q3)	1936.77 (1405.5, 3040.56)	1988.6 (1443.68, 3109.17)	1873.18 (1370.14, 2970.82)	
Q1	2841 (25.0)	1322 (23.1)	1520 (26.9)	< .0001
Q2	2842 (25.0)	1393 (24.4)	1449 (25.6)	
Q3	2843 (25.0)	1521 (26.6)	1321 (23.4)	
Q4	2841 (25.0)	1475 (25.8)	1366 (24.2)	

*KM* Korean medicine, *PELD* percutaneous endoscopic lumbar decompression, *Pt_plasty* percutaneous plasty, *C_plasty* cervical spine laminoplasty.

### KM expenditure and the time to the first use of KM

Table 3 presents the KM expenditure and the time to the first use of KM according to the basic characteristics of participants and index surgery. In the KM use group, the median cost for all KM expenses was $172.32 (IQR: 57.85–504.02), and the time to the first use of KM was 356 (IQR: 109–840) days. The KM expenditure was higher and the time to the first use of KM was shorter in female patients, elderly patients, participants with higher CCI scores, and those who had experience with KM use prior to the surgery. A similar tendency was shown when compared with the average expenditure and time (Supplementary Table S4). Meanwhile, Supplementary Table S5 shows KM treatment details and costs received by patients after surgery.

**Table 3:** KM expenditure and the time to the first use of KM.

	KM expenditure (US dollars)		Time to the first use of KM (days)	
	Median	*p*-value	Median	*p*-value
Total (N = 5711)	172.32 (57.85, 504.02)		356 (109, 840)	
Sex				
Male	147.76 (49.45, 397.13)	< .0001	383 (115, 868.5)	0.019
Female	194.12 (66.26, 557.81)		343 (106, 811)	
Age				
0–19	116.99 (47.02, 349.6)	< .0001	635 (143, 1133)	< .0001
20–29	66.43 (27.29, 195.45)		619 (279, 1086)	
30–39	98.12 (33.2, 292.53)		486.5 (174.5, 1103)	
40–49	123.56 (45.18, 328.76)		369 (112, 860)	
50–59	148.59 (54.22, 427.8)		393 (115, 879)	
60–69	192.28 (69.36, 573.67)		379 (126, 854)	
≥ 70	236.19 (84.15, 620.38)		286 (88, 696)	
Income				
Level 1 (low)	183.94 (66.17, 507.72)	0.1255	349 (109, 821)	0.31
Level 2	165.18 (53.5, 470.03)		366 (122, 889)	
Level 3	160.3 (53.1, 500.83)		364 (112.5, 840.5)	
Level 4	180.07 (60.2, 520.57)		348 (100, 800)	
Region				
Seoul	178.71 (56.47, 513.19)	0.002	388 (127, 861)	< .0001
Capital area	158.15 (52.82, 463.86)		410 (125, 924)	
Metropolitan city	191.2 (67.66, 572.76)		331 (102, 811)	
Others	172.19 (58.77, 502.6)		328 (97, 781)	
CCI^3^				
0	117.81 (41.21, 342.35)	< .0001	439 (154, 977.5)	< .0001
1	143.16 (51.83, 414.24)		385 (109, 886)	
2	167.44 (59.39, 488.98)		365 (117, 853)	
3	220.11 (70.59, 573.67)		299 (95, 694)	
≥ 4	245.36 (86.56, 642.14)		300 (93, 759)	
Use of KM before surgery				
None	128.5 (47.16, 355.07)	< .0001	501 (183, 980)	< .0001
KM use	221.21 (74.95, 622.71)		260 (80, 666)	
Type of surgery				
Fusion	175.34 (62.17, 519.89)	0.1121	405.5 (135, 847.5)	0.0024
Open discectomy	160.03 (53.45, 459.45)	< .0001	395.5 (129.5, 853)	< .0001
PELD	119.32 (50.49, 333.27)	0.199	358 (128, 771)	0.5956
Nucleolysis	308.83 (184.46, 638.9)	0.0923	362 (156, 682.5)	0.9444
Pt_plasty	208.14 (74.78, 617.67)	< .0001	243 (75, 705)	< .0001
Laminectomy	214.07 (68.93, 559.78)	< .0001	357 (108, 867)	0.9153
C_plasty	206.24 (56.73, 534.3)	0.7756	229.5 (77, 747)	0.3417
Corpectomy	377.53 (144.35, 830.74)	0.036	318.5 (93.5, 861.5)	0.7913
Reduction	99.66 (25.1, 237.01)	0.0562	163 (38, 606)	0.0853
Others	277.18 (103.7, 737.09)	0.1371	281.5 (58, 533)	0.2867
Complexity of surgery				
Percutaneous	199.2 (71.4, 567.94)	< .0001	251 (78, 705)	< .0001
Open (simple)	163.12 (52.71, 459.45)		372 (117, 874.5)	
Open (with instrument)	175.34 (62.17, 519.89)		405.5 (135, 847.5)	
Types of disease				
Lumbar disc disorders	150.41 (50.59, 427.06)	< .0001	388 (125, 869)	< .0001
Fracture	199.19 (71.07, 564.81)		258 (78, 709)	
Stenosis	215.03 (79.8, 581.81)		366.5 (121, 854)	
Deforming dorsopathies	174.12 (52.24, 534.99)		404 (146, 824)	
Cervical disc disorders	120.07 (47.89, 388.07)		428 (109, 881.5)	
Spondylosis	149.47 (52.16, 324)		353 (86, 721)	
Inflammatory spondylopathies	264.12 (67.16, 516.87)		746 (493, 1044)	
Dorsalgia	209.3 (88.32, 534.31)		249 (90, 616)	
Dorsopathy	97.71 (38.46, 261.11)		763 (308, 945)	
Other spondylopathies	182.52 (53.21, 485.48)		177 (56, 623)	
Disease sites				
Cervical spine	128.02 (50.15, 385.65)	0.0033	450 (109, 871)	0.0003
Thoracic spine	197.46 (78.62, 544.3)		262.5 (83, 664)	
Lumbar spine	175.06 (57.97, 505.74)		362.5 (114, 844)	
Others	166.61 (78.79, 470.94)		268.5 (96.5, 788.5)	
Type of institutions				
Clinic	208.07 (63.91, 673.27)	0.5581	243.5 (61, 802)	0.042
Hospital	170.34 (57.7, 499.39)		361 (107, 850)	
General hospital	165.68 (54.66, 515.16)		343 (105, 797.5)	
Tertiary hospital	178.24 (62.76, 466.97)		387.5 (139, 838)	

*CCI* Charlson Comorbidity Index, *KM* Korean medicine, *PELD* percutaneous endoscopic lumbar decompression, *Pt_plasty* percutaneous plasty, *C_plasty* cervical spine laminoplasty.

### Factors affecting the use of KM after spinal surgery

A two-part model was used to analyze the factors affecting the use of KM and the cost of using KM after spinal surgery. The univariate analysis revealed that female sex, older age, CCI score, and prior experience with KM use were factors that increased the use and medical costs of KM after surgery. Patients living in metropolitan cities and other areas had higher odds of using KM, whereas those living in capital areas had lower odds of using KM and lower medical costs for KM (Table 4).Spinal stenosis and a higher than median cost of spinal surgery were associated with greater odds of using KM and increased KM expenditure.

**Table 4:** Results of univariate analysis for factors affecting the use of KM after spinal surgery.

	Probability (logit)	Conditional (GLM)		Marginal effects
	OR (95% CI)	Coeff (SE)	*p*-value	Margin (95% CI)
Sex (ref = male)				
Male				180.95 (165.74 to 196.16)
Female	1.53 (1.42 to 1.65)	0.23 (0.05)	0	280.69 (261.96 to 299.43)
Age (ref = < 30)				
< 30				159.90 (141.05 to 178.75)
30–50	1.31 (1.17 to 1.46)	0.15 (0.08)	0.059	215.45 (189.36 to 241.54)
50–70	1.82 (1.63 to 2.03)	0.28 (0.08)	0	285.99 (254.63 to 317.36)
≥ 70	1.61 (1.46 to 1.77)	0.31 (0.07)	0	278.05 (252.52 to 303.58)
Income (ref = level 1, low)				
Level 1 (low)				253.04 (225.19 to 280.88)
Level 2	0.92 (0.82 to 1.02)	− 0.06 (0.07)	0.444	229.15 (204.49 to 253.82)
Level 3	1.01 (0.90 to 1.12)	− 0.05 (0.07)	0.516	241.92 (216.25 to 267.59)
Level 4	1.02 (0.92 to 1.13)	− 0.12 (0.07)	0.092	227.45 (206.01 to 248.90)
Region (ref = Seoul)				
Seoul				243.85 (210.80 to 276.90)
Capital area	0.98 (0.87 to 1.10)	− 0.22 (0.08)	0.005	192.84 (173.43 to 212.24)
Metropolitan city	1.30 (1.15 to 1.48)	0.06 (0.09)	0.45	296.56 (262.08 to 331.05)
Others	1.25 (1.12 to 1.40)	− 0.14 (0.08)	0.063	237.10 (217.92 to 256.27)
CCI (ref = 0)				
0				162.37 (140.98 to 183.76)
1	1.32 (1.18 to 1.48)	0.04 (0.08)	0.591	197.60 (175.88 to 219.31)
2	1.48 (1.32 to 1.66)	0.14 (0.08)	0.086	231.03 (204.32 to 257.73)
3	1.81 (1.59 to 2.06)	0.26 (0.09)	0.003	286.07 (247.76 to 324.38)
≥ 4	1.77 (1.58 to 1.99)	0.39 (0.08)	0	320.99 (286.57 to 355.41)
Use of KM before surgery (ref = none)				
None				147.23 (135.32 to 159.14)
KM use	2.93 (2.71 to 3.16)	0.35 (0.05)	0	351.29 (327.14 to 375.44)
Type of surgery (ref = decom)				
Decom (n = 4724)				208.58 (191.50 to 225.65)
Fusion (n = 3025)	1.07 (0.97 to 1.17)	0.18 (0.06)	0.003	258.63 (232.64 to 284.62)
Pt-plasty (n = 2340)	0.98 (0.88 to 1.08)	0.20 (0.07)	0.003	251.70 (222.23 to 281.17)
Others (n = 1278)	1.31 (1.16 to 1.49)	0.08 (0.08)	0.3	257.95 (220.21 to 295.69)
Complexity of surgery (ref = percutaneous)				
Percutaneous				243.88 (216.30 to 271.47)
Open (simple)	1.09 (0.99 to 1.19)	− 0.14 (0.06)	0.031	221.57 (205.60 to 237.55)
Open (with an instrument)	1.09 (0.98 to 1.21)	0.01 (0.07)	0.837	258.63 (232.73 to 284.53)
Types of disease (ref = lumbar disc disorders)				
Lumbar disc disorders				208.70 (161.33 to 226.07)
Fracture	0.51 (0.84 to 1.03)	0.15 (0.07)	0.026	234.20 (207.15 to 261.24)
Stenosis	1.38 (1.25 to 1.52)	0.18 (0.07)	0.006	290.81 (259.42 to 322.20)
Deforming dorsopathies	1.13 (0.98 to 1.31)	0.27 (0.10)	0.005	289.52 (236.54 to 342.50)
Cervical disc disorders	0.89 (0.76 to 1.03)	0.00 (0.11)	0.993	196.05 (155.42 to 236.68)
Others	0.96 (0.77 to 1.19)	0.41 (0.15)	0.782	212.59 (148.45 to 276.73)
Disease sites (ref = cervical spine)				
Cervical spine				191.09 (156.28 to 225.90)
Thoracic spine	1.20 (1.01 to 1.42)	0.17 (0.12)	0.149	249.81 (206.30 to 293.32)
Lumbar spine	1.28 (1.13 to 1.46)	0.10 (0.09)	0.277	240.11 (226.23 to 254.00)
Others	1.16 (0.83 to 1.61)	0.12 (0.23)	0.6	232.98 (130.03 to 335.94)
Type of institutions (ref = clinic)				
Clinic				251.55 (172.64 to 330.46)
Hospital	1.03 (0.82 to 1.29)	− 0.03 (0.15)	0.834	246.98 (230.53 to 263.42)
General hospital	0.85 (0.67 to 1.07)	− 0.09 (0.16)	0.583	212.07 (189.39 to 234.75)
Tertiary hospital	0.92 (0.72 to 1.18)	− 0.04 (0.16)	0.825	232.57 (199.03 to 266.11)
Costs of surgery (ref = Q1)				
Q1				193.85 (172.90 to 214.81)
Q2	1.11 (1.00 to 1.23)	− 0.03 (0.07)	0.726	198.92 (178.03 to 219.81)
Q3	1.32 (1.19 to 1.47)	0.20 (0.07)	0.004	273.14 (245.86 to 300.42)
Q4	1.24 (1.12 to 1.38)	0.26 (0.07)	0	279.38 (250.98 to 307.78)
Length of stay (ref = Q1)				
Q1				198.87 (177.31 to 220.43)
Q2	1.06 (0.96 to 1.18)	− 0.05 (0.07)	0.476	194.88 (173.79 to 215.96)
Q3	1.23 (1.11 to 1.37)	0.11 (0.07)	0.132	245.52 (221.31 to 269.73)
Q4	1.26 (1.14 to 1.40)	0.30 (0.07)	0	301.79 (271.68 to 331.90)

*KM* Korean medicine, *GLM* generalized linear model, *Decom* decompression surgery, *Pt-plasty* percutaneous plasty, *OR* odds ratio, *SE* standard error, *CI* confidence interval, *CCI* Charlson Comorbidity Index.

The final model was determined by analyzing the results of the univariate and multivariate models with all variables included in the univariate analysis (Table 5). Female sex, older age, high CCI score, and prior experience with KM use were factors that increased KM use and expenditure after surgery. The odds for using KM were higher among those living in metropolitan cities. A similar trend was observed in the sensitivity analysis results, in which the use of KM was defined as within 1 year or 3 years instead of 5 years (Tables S6 and S7).

**Table 5:** Factors affecting the use of KM after spinal surgery: the final model.

	Probability (logit)	Conditional (GLM)		Marginal effects
	OR (95% CI)	Coeff (SE)	*p*-value	Margin (95% CI)
Sex (ref = male)				
Male				192.01 (175.34 to 208.67)
Female	1.44 (1.33 to 1.57)	0.18 (0.05)	0.001	269.04 (250.86 to 287.22)
Age (ref = < 30)				
< 30				197.74 (171.77 to 223.70)
30–50	1.20 (1.06 to 1.35)	0.03 (0.08)	0.711	220.98 (194.36 to 247.60)
50–70	1.53 (1.35 to 1.74)	0.09 (0.08)	0.265	260.40 (232.81 to 287.98)
≥ 70	1.42 (1.25 to 1.62)	0.10 (0.08)	0.232	255.24 (230.36 to 280.12)
Region (ref = Seoul)				
Seoul				253.76 (218.47 to 289.05)
Capital area	1.03 (0.91 to 1.16)	− 0.24 (0.08)	0.004	202.29 (181.49 to 223.09)
Metropolitan city	1.29 (1.13 to 1.48)	0.04 (0.09)	0.633	294.89 (259.46 to 330.31)
Others	1.22 (1.09 to 1.38)	− 0.20 (0.08)	0.011	226.93 (208.39 to 245.48)
CCI (ref = 0)				
0				192.25 (166.32 to 218.18)
1	1.22 (1.09 to 1.38)	0.01 (0.08)	0.865	213.27 (189.95 to 236.59)
2	1.26 (1.11 to 1.43)	0.07 (0.08)	0.417	228.29 (202.40 to 254.18)
3	1.45 (1.26 to 1.67)	0.13 (0.09)	0.161	256.69 (222.92 to 290.45)
≥ 4	1.37 (1.20 to 1.55)	0.27 (0.08)	0.002	287.39 (256.63 to 318.14)
Use of KM before surgery (ref = none)				
None				151.97 (139.57 to 164.36)
KM use	2.82 (2.60 to 3.05)	0.34 (0.05)	0.000	342.92 (319.27 to 366.58)
Type of surgery (ref = decom)				
Decom (n = 4724)				210.81 (184.08 to 237.54)
Fusion (n = 3025)	0.98 (0.84 to 1.15)	0.09 (0.10)	0.392	228.40 (195.46 to 261.33)
Pt-plasty (n = 2340)	0.98 (0.74 to 1.29)	0.45 (0.18)	0.015	327.17 (227.00 to 427.35)
Others (n = 1278)	1.15 (0.98 to 1.35)	0.05 (0.10)	0.634	233.30 (194.58 to 272.02)
Types of disease (ref = lumbar disc disorders)				
Lumbar disc disorders				272.05 (228.59 to 315.50)
Fracture	0.77 (0.59 to 1.00)	− 0.38 (0.18)	0.032	165.57 (124.83 to 206.31)
Stenosis	1.05 (0.91 to 1.21)	− 0.01 (0.09)	0.946	275.87 (233.70 to 318.05)
Deforming dorsopathies	0.88 (0.72 to 1.07)	0.02 (0.12)	0.847	264.30 (205.32 to 323.28)
Cervical disc disorders	1.07 (0.88 to 1.29)	− 0.06 (0.13)	0.635	262.59 (199.32 to 325.86)
Others	0.85 (0.66 to 1.09)	− 0.21 (0.16)	0.194	205.41 (143.63 to 267.20)
Costs of surgery (ref = Q1)				
Q1				231.72 (197.79 to 265.64)
Q2	0.97 (0.86 to 1.10)	− 0.04 (0.08)	0.603	219.54 (193.27 to 245.82)
Q3	1.07 (0.93 to 1.23)	0.06 (0.09)	0.509	253.20 (225.96 to 280.45)
Q4	0.99 (0.83 to 1.17)	0.04 (0.11)	0.722	239.93 (207.18 to 272.68)
Length of stay (ref = 0)				
Q1				215.53 (186.57 to 244.48)
Q2	1.01 (0.89 to 1.14)	− 0.02 (0.08)	0.842	212.78 (188.41 to 237.14)
Q3	1.05 (0.92 to 1.20)	0.08 (0.09)	0.384	237.82 (213.22 to 262.42)
Q4	1.05 (0.91 to 1.22)	0.21 (0.10)	0.027	272.19 (240.92 to 303.45)

*KM* Korean medicine, *GLM* generalized linear model, *CI* confidence interval, *Decom* decompression surgery, *Pt_plasty* percutaneous plasty, *SE* standard error, *CCI* Charlson Comorbidity Index.

## Discussion

With spinal surgery and surgical costs increasing worldwide^7–10^, many patients in Korea use KM after spinal surgery^24,25^. Although several studies have been conducted to show the effectiveness of KM treatments, including acupuncture^12,26^, electroacupuncture^27–29^, and moxibustion^30^, no study has investigated the KM use status in patients after spinal surgery.

The present study had a large sample size (N = 11,367 patients) and used nationally representative data. In this study, female sex, older age, high CCI score, and the use of KM before surgery were identified as factors associated with KM use and expenditure. The probability of using KM was high when living in areas with low medical expenses other than Seoul. In addition, in the case of plastic surgery and surgery for stenosis, KM medical expenses were high. Several studies analyzing the general determinants of KM use have reported a high proportion of female and elderly participants in the KM use group^31–33^. Women tend to accept traditional medicine better than men and seek it more frequently^34,35^. Women and the elderly in Korea often have relatively low socioeconomic status, several chronic diseases, and low subjective health status. According to statistics from Ministry of employment and labor In Korea, the total monthly wage was 2,645,000 Korean won for over years old. This is lower than 3,710,000 in 30 s, 4,195,000 in 40 s, and 3,909,000 in 50 s. By sex, monthly wages were 4,127,000 for male and 2,683,000 for female^36^. Therefore, the demand for medical care is high; however, there is a high possibility of unmet medical care^37–39^. The results of this study suggest that KM may be considered a means to satisfy the unmet medical needs of women and the elderly after surgery.

The median days until first medical use after surgery for KM users was 8 days, while the median days until first KM use was 365 days. In other words, KM was used about a year after surgery, which was later than using conventional treatment. This suggested that patients tend to use conventional treatment in the early stages after surgery, but seek KM when symptoms become chronic and do not approved. The CCI score, surgical cost, and hospitalization days were higher in the KM use group, suggesting that patients with relatively severe diseases used KM more often. In particular, the proportion of patients who underwent surgery for stenosis in the KM use group was high, which reflects the recent rapid increase in the use of KM for stenosis. According to the health insurance statistics of the NHIS, the number of patients using KM for stenosis in 2010 increased by approximately three and a half times from 38,090 to 134,337 in 2020. This is a significant increase compared with the 1.8-fold increase in the number of patients with stenosis within the same period. However, only few studies on the effects of KM on stenosis have been conducted, and additional research on this topic is required.

Prior experience with KM use was the most influential factor for the use of KM after surgery. In other words, patients who received KM treatment were more likely to continue to choose KM. Several factors influence the patients’ choice of treatment. In an interview study conducted by Kim et al.^40^, personal experiences and advice from people in one’s network were shown to be important factors in choosing a treatment method. These factors particularly influenced the decision to choose the KM treatment. Patients who directly or indirectly experienced the effects of KM treatment had a positive perception of KM and chose KM rather than surgery. Patel et al.^41^ revealed through interviews that individuals' positive experiences and word-of-mouth about acupuncture had an impact on the pursuit of traditional Chinese medicine. Another study^42^ found that when choosing a primary care physician or specialist, information or recommendations from acquaintances were more important than other factors.

These individual and community experiences often lead to the formation of medical beliefs, which affect treatment selection and medical service usage patterns more than any other objective factors^43,44^. With respect to the medical beliefs that lead to the selection of KM, KM is believed to be superior and to have fewer side effects with less toxicity, in addition to the recognition of limitations in drug treatment^41,45,46^. The high rate of KM use after surgery in patients with KM experience means that past KM experience helped form these positive medical beliefs about KM. In addition, the longer the treatment period, the more patients learn about and try various treatments. As a result, patients may be reminded of past experiences they had forgotten, and there is a possibility that they will use KM as they collect more information about various treatments and stories from acquaintance. Accordingly, a long treatment period may be a factor that increases KM use rate, but this study was not able to isolate this factor.

Ultimately, this study revealed that patient characteristics (particularly, previous experience in using KM), rather than surgical-related characteristics, affected the use of KM after surgery. This finding was similar to the factors affecting the general use of KM.

Meanwhile, KM treatment generally consists of acupuncture, electroacupuncture, pharmacopuncture, herbal medicine, and Chuna manual therapy. There are several studies showing that such KM treatment has the potential to be an effective treatment for improving pain and function in patients after spine surgery. In a study in which 16 weeks of KM treatment was conducted on 120 patients with persistent postoperative pain^26^, the visual analogue scale (VAS) for back and lower extremity pain improved from 6.1 to 2.9 and from 5.4 to 2.4 respectively after 6 months. In one systematic review^12^, it was found that acupuncture treatment 24 h after surgery showed a positive effect on pain reduction compared to sham treatment. Another study comparing the usual care and electroacupuncture combination with the usual care in patients with pain after spine surgery, the combination treatment with electroacupuncture was more effective and cost-effective in improving VAS and Oswestry disability index.

This study had some limitations. The data utilized for the analyses were obtained from the NHIS-NSC database, and only information from this database could be analyzed. The data provided by the sample cohort were based on claims submitted by medical institutions for reimbursement; therefore, non-covered items were not included. Furthermore, surgery and KM have a high proportion of non-covered items. Hence, the cost calculated in this study might have underestimated the actual cost incurred in the real world. However, since patients rarely receive only non-covered treatment, it is very unlikely that there will be bias in the results of KM use analysis.

In addition, diagnosed diseases and types of surgery were described using disease and procedure codes only. Other potentially important factors, such as operative time, amount of transfused blood, and degree of pain before and after surgery, were not considered because of data limitations. If these factors had been investigated, a more diverse and rich analysis of the factors affecting KM use would have been possible. However, despite the limitations of the data, we extracted as many surgery-related outcomes as possible through various operational definitions such as surgery complexity and disease type, and also extracted as many variables as possible for patient characteristics. Additional studies that thoroughly describe the characteristics of surgery using surgery-related outcome variables are needed.

It is also unfortunate that clinical outcome data such as VAS and ODI could not be analyzed due to database limitations. If such data were available, it would have been possible to analyze changes in outcomes according to KM use. In the future, research can be conducted to examine changes in outcomes according to the use of KM by combining database and hospital data.

Nevertheless, this study is the first to analyze the use of KM after spinal surgery in detail. In particular, all types of spinal surgery were analyzed, and this study was not limited to specific widely studied surgeries, such as stenosis and lumbar disc herniation, to provide a comprehensive view of the use of KM after spinal surgery. In addition, the effects of various patient and surgical characteristics on the use of KM were analyzed through various operational definitions, and the belief formed through experience in using KM was considered as a major factor influencing the health-seeking behavior of using KM. The results of this study provide useful information to KM clinicians who treat patients in real world and policymakers who establish policies related to coverage and accessibility of KM through a deep understanding of patients who use KM after surgery.

In conclusion, female sex, older age, high CCI score, and prior experience with KM use were factors that increased KM use and expenditure after surgery. Additional research can be conducted by combining other variables that cannot be extracted from this database with the database. The results of this study may be useful to clinicians, and policymakers.

## Methods

### Study design

This retrospective cohort study included patients who underwent spinal surgery for spinal diseases from 2011 to 2014 and were followed up for 5 years. For these patients, medical care for spinal diseases was investigated. In addition, patients’ and spinal surgery characteristics, use and costs of KM after spinal surgery, and factors associated with the use of KM were analyzed. This study was reviewed and qualified as an exemption by the Institutional Review Board of Kyung Hee University (approval no.: KHSIRB-22-261, approval date: 31 May 2022). The principles expressed in the Declaration of Helsinki were adhered to in the study’s analysis. As the study analyzed publicly available data, it was exempted from the consent process by the Institutional Review Board of Kyung Hee University. All personal information was de-identified by the NHIS prior to public release.

### Data source

Data from the NHIS-NSC 2.2 database from 2011 to 2019 were analyzed in this study. Korea has a nationwide medical insurance system; hence, most people are enrolled in the National Health Insurance Service (NHIS). In the case of covered items, medical costs are paid by the NHIS and the patient. For reimbursement of the co-payment, each medical institution submits insurance claims to the NHIS containing detailed information about the service provided. Accordingly, the NHIS contains data related to the use of medical care by the entire population. The NHIS established and used a sample cohort with a stratified sampling of the entire population for research purposes^47^. The NHIS-NSC 2.2 database comprises sample data from a randomly selected and stratified sample of 2.2% of the Korean population in 2006 who were followed up until 2019.

### Participants

**Figure 2:**
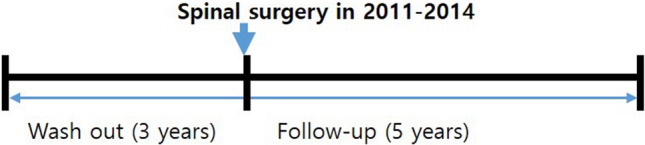
A brief summary of the study design.

This retrospective cohort study included patients who underwent spinal surgery for back pain and spinal diseases between 2011 and 2014 (Fig. 2). Patients with infections, parasitic diseases, malignant/benign neoplasms, or fractures were excluded. Moreover, patients with spinal tuberculosis, brucella spondylitis, enterobacterial spondylitis, and spondylopathy were excluded. In addition, patients with a history of spinal surgery within 3 years before the first surgery between 2011 and 2014 were excluded. Therefore, patients who underwent surgery for the first time were included. Spinal diseases were defined based on the disease names of the 7th Korean Standard Classification of Disease (KCD-7) and 10th International Standard Classification of Disease (ICD-10). The inclusion and exclusion KCD-7 codes are listed in Supplementary Table S1. Lastly, patients who used KM at least once within the 5 years of follow-up were assigned to the KM use group, whereas those who did not use KM were assigned to the non-KM use group.

### Variables

#### Index surgery

The index date for spinal surgery was defined as the hospitalization date of the index hospitalization during which spinal surgery was performed. In Korea, even one hospitalization episode can cause multiple claims for various reasons; therefore, the definition of index hospitalization episodes should be carefully considered. In this study, based on claims that included surgery, connected claims with a hospitalization date with a 1-day interval from the discharge date were considered as one hospitalization episode. However, hospitalization episodes were excluded (1) in cases of outpatient procedures, (2) when the disease codes in the connected claims were not disease inclusion codes, and (3) when the institution was different from the institution where the surgery was performed.

**Figure 3:**
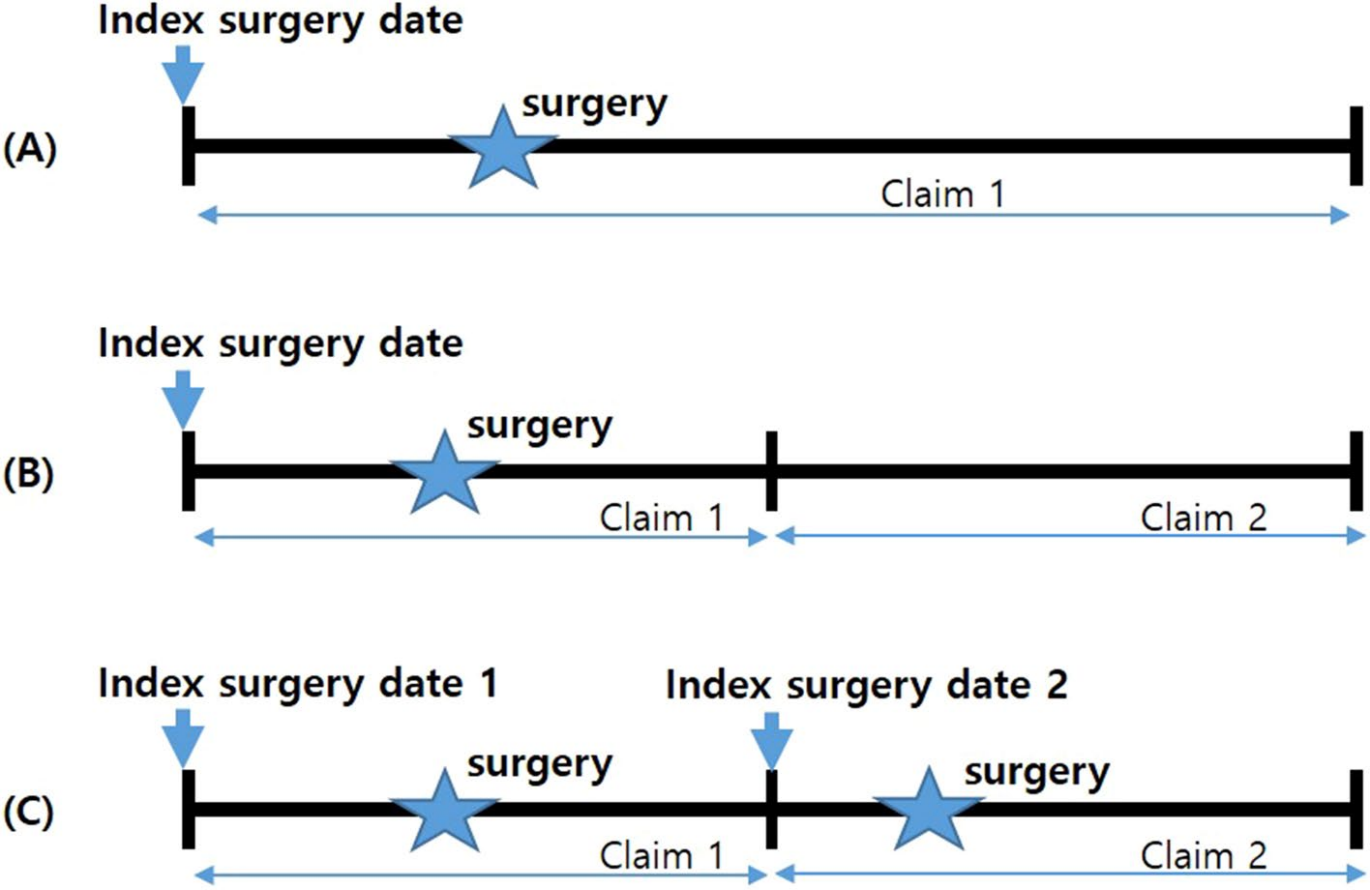
Index surgery date and hospitalization episode. (**A**) No connected claim; one claim was considered as one hospitalization episode. (**B**) If there were two connected claims with no surgical records, the two claims were regarded as one hospitalization episode, and the admission date for the first claim was set as the index surgery date. (**C**) If there were connected claims with surgical records, each claim was regarded as an independent hospitalization episode, and the admission date for each claim was set as the index date.

In addition, if there was another spinal surgery in the connected claims, the claim was defined as another hospitalization episode and considered a reoperation. Claims that occurred in the same institution during hospitalization were added to the index hospitalization episode (Fig. 3).

#### Characteristics of spinal surgery

The characteristics of spinal surgery, including type of surgery, complexity of surgery, type of disease, disease area, type of institution, length of hospital stay, and cost of surgery were investigated. The types of surgery were classified as fusion, open discectomy, percutaneous endoscopic lumbar decompression (PELD), nucleolysis, percutaneous plasty, laminectomy, cervical spine laminoplasty, corpectomy, and reduction. The complexities of surgery were classified as non-invasive, open surgery, or open surgery with an instrument. If multiple surgical codes were applied in one hospitalization episode, duplication was allowed for the types of surgery and included in the classification of the complexity of surgery. For convenience, the type of surgery was classified during factor analysis as decompression (discectomy, laminectomy, or no fusion), fusion, percutaneous plasty (only percutaneous plasty), and others. All spinal surgeries were defined based on procedure codes in the claims. The codes were selected based on previous studies^24,48^. The procedure codes for the classification of types and complexities of surgery are presented in Supplementary Table S2.

Disease types were classified as lumbar disc disorder, fracture, stenosis, deforming dorsopathy, cervical disc disorders, spondylosis, inflammatory spondylopathies, dorsalgia, dorsopathy, and other spondylopathies. For factor analysis, spondylosis, inflammatory spondylopathies, dorsalgia, dorsopathy, and other spondylopathies were grouped because of their low frequency. Disease areas were classified as cervical, thoracic, and lumbar spines (Supplementary Table S3). All classifications were based on the KCD-7. Surgical institutions were classified into tertiary general hospitals, general hospitals, hospitals, and clinics, according to the national classification criteria. The classification criteria for spinal surgery were decided through an internal meeting of the research team.

#### Patient characteristics

Variables related to patient characteristics were selected based on the Andersen healthcare utilization model^49^ and information available from this database. The Andersen model is the most basic model that explains patients' medical utilization. In this study, sex, age, area of residence, prior experience with KM use were included as predisposing factors, income level as enabling factors, and CCI as necessary factors. Age was classified as < 20, 20–29, 30–39, 40–49, 50–59, 60–69, and > 70 years old. Income was categorized into four stages by reclassifying the 10th decile classification in the data. Residential areas were classified as Seoul, capital areas, metropolitan cities, and others. The capital areas included Gyeonggi-do and Incheon, whereas the metropolitan cities included Daejeon, Daegu, Gwangju, Ulsan, and Busan. CCI was calculated based on all diseases that occurred in the year before the index date and was classified as 0, 1, 2, 3, and 4 points or more. In addition, the use of KM for spinal diseases during the year before the index date was included as a variable.

#### Use of KM

The use of KM within the follow-up period, the total cost of KM treatment, and the time until the first use of KM were determined.

### Statistical analysis

Descriptive statistics were calculated to describe the characteristics of patients and index surgery in the KM and non-KM use groups. Continuous variables are presented as mean and standard deviation (SD), whereas categorical variables are expressed as frequency and percentage. The differences between the two groups were analyzed using the chi-square test and independent t-test.

Medical expenditures for KM use and the time until the first use of KM according to the basic characteristics of patients and index surgery in the KM use group are presented as medians and interquartile ranges. Differences in characteristics were examined using the Wilcoxon rank-sum test.

A two-part model^50,51^ was used to analyze factors affecting the use of KM in patients who underwent spinal surgery. This technique is used for semi-continuous data with abundant zero values, such as medical expenditure^52^. In a two-part model, analysis consists of a process of determining the probability of a positive value occurring and a process of predicting the positive value. In this study, the prediction of the mean dependent variable and KM expenditure also depends on two factor s. The probability of patients using KM was modeled in the first part of the two-part model. In the second part, the main concern was to predict medical expenditure by patients using KM.

For the first part, of the possible logit and probit models, the logit model was used because it is widely used for the analyses of this nature. The difference between the results of the two models is not significant; thus, either model may be used^51^. In the second part, the generalized linear model (GLM) requires choosing a link function and a distribution family. First, the Box-Cox test was used to determine the link function. In this study, all univariate and multivariate models showed a scalar power close to 0; therefore, the natural log link function was selected^53^. Next, gamma distribution was used for the distribution family. The gamma distribution is a flexible distribution that can be used when analyzing continuous, positively skewed, and positive data^54^. Therefore, GLM with gamma distribution is commonly used to analyze data with mass zeros and right-skewed distribution, such as medical expenditure^55^.

The final model was determined by referring to the results of the univariate models and multivariate model including all variables used in univariate models. For the univariate model, the following variables were analyzed; sex, age, income, region, CCI, Use of KM before surgery, type of surgery, complexity of surgery, types of disease, disease sites, type of institutions, costs of surgery, length of stay. The results are presented with odds ratios (OR) and 95% confidence intervals (95% CI) in the first part and coefficient and robust standard error (SE) in the second part.

In addition, the marginal effects for each variable are shown with a 95% CI. Marginal effects are an alternative way to express results that can add an intuitive understanding to the meaning of the analysis. They measure the effect of a one-unit change in a specific explanatory variable on the conditional mean of a dependent variable. In other words, the marginal effect of a specific variable indicates changes in the result due to changes in a specific explanatory variable when all other explanatory variables are fixed at the average^56^.

Medical expenditures were calculated in Korean won (KRW) and converted to the United States dollars using the average exchange rate from 2011 to 2019 (1119.11 KRW/USD). All statistical analyses were performed using SAS version 9.4 (SAS Institute Inc., Cary, NC, USA) and Stata 17 (StataCorp, College Station, TX, USA) with the “twopm” command.

### Ethics statement

The current study was reviewed and qualified as an exemption by the Institutional Review Board of Kyung Hee University (KHSIRB-22-261). The principles expressed in the Declaration of Helsinki were adhered to in the study’s analysis. As the study analyzed publicly available data, no consent was obtained from patients; all personal information was de-identified by the NHIS prior to public release.

### Supplementary Information

Supplementary Information.

## Data Availability

This study incorporated data from the National Health Insurance Service Sample Cohort 2.2 (2002–2019), released by the Korean National Health Insurance Service in response to the researchers’ request. The detailed cohort profile and methods for obtaining data are explained in the following sources: Lee J, Lee JS, Park SH, Shin SA, Kim K. Cohort Profile: The National Health Insurance Service-National Sample Cohort (NHIS-NSC), South Korea. International Journal of Epidemiology. 2016. https://doi.org/10.1093/ije/dyv319. PubMed PMID:26822938.
