# Supplementary material for: Analysis of factors associated with the use of Korean medicine after spinal surgery using a nationwide database in Korea
Source: Sci Rep. 2023 Nov 17;13:20177. doi: 10.1038/s41598-023-47454-5 (PMC10656548; doi:10.1038/s41598-023-47454-5)
Supplement: Supplementary file 1 — Supplementary Information. [file 41598_2023_47454_MOESM1_ESM.docx]

**Factors associated with the use of Korean medicine after spinal surgery: A nationwide retrospective cohort study**

**Short title:** Korean medicine use after spinal surgery in Korea

Doori Kim**^1,2^**^¶^, Yoon Jae Lee**^2^**^¶^, Bo-Hyoung Jang^1^*, Jeong-Su Park^3^, Sunju Park^4^, Christopher R D’Adamo^5^, Yong Cheol Shin^1^ & Seong-Gyu Ko^1^

^1^Department of Preventive Medicine, College of Korean Medicine, Kyung Hee University, 26 Kyungheedae-ro, Dongdaemun-gu, Seoul 02447, Republic of Korea

^2^Jaseng Spine and Joint Research Institute, Jaseng Medical Foundation, 540 Gangnam-daero, Gangnam-gu, Seoul 06110, Republic of Korea

^3^Department of Preventive Medicine, College of Korean Medicine, Semyung University, 65, Semyeong-ro, Jecheon-si, Chungcheongbuk-do, Republic of Korea

^4^Department of Preventive Medicine, College of Korean Medicine, Daejeon University, 62, Daehak-ro, Dong-gu, Daejeon 34520, Republic of Korea

^5^Department of Family & Community Medicine, University of Maryland School of Medicine, 655 West Baltimore Street, Baltimore, MD 21201, USA

^¶^These authors contribute equally to this work.

***Correspondence:** Bo-Hyoung Jang, Department of Preventive Medicine, College of Korean Medicine, Kyung Hee University, 26 Kyungheedae-ro, Dongdaemun-gu, Seoul 02447, Republic of Korea. Tel: +82-2-961-2219; Fax: +82-2-961-238; Email: [bhjang@khu.ac.kr](mailto:bhjang@khu.ac.kr)

**Supplementary Table S1.** Inclusion and exclusion disease codes

| **KCD-7** | **Disease name** |
| --- | --- |
| **Inclusion codes** | |
| M40 | [Kyphosis](http://wikipedia.org/wiki/Kyphosis) and [lordosis](http://wikipedia.org/wiki/Lordosis) |
| M41 | Scoliosis |
| M42 | [Spinal osteochondrosis](http://wikipedia.org/wiki/Spinal_osteochondrosis) |
| M43 | Other deforming [dorsopathies](http://wikipedia.org/wiki/Dorsopathies) |
| M45 | [Ankylosing spondylitis](http://wikipedia.org/wiki/Ankylosing_spondylitis) |
| M46 | Other [inflammatory](http://wikipedia.org/wiki/Inflammation) [spondylopathies](http://wikipedia.org/wiki/Spondylopathies) |
| M47 | [Spondylosis](http://wikipedia.org/wiki/Spondylosis) |
| M48 | Other [spondylopathies](http://wikipedia.org/wiki/Spondylopathies) |
| M49 | [Spondylopathies](http://wikipedia.org/wiki/Spondylopathies) in [diseases](http://wikipedia.org/wiki/Diseases) classified elsewhere |
| M50 | [Cervical disc disorders](http://wikipedia.org/wiki/Cervical_disc_disorders) |
| M51 | Other [intervertebral disc disorders](http://wikipedia.org/wiki/Intervertebral_disc_disorders) |
| M53 | Other [dorsopathies](http://wikipedia.org/wiki/Dorsopathies), NEC |
| M54 | [Dorsalgia](http://wikipedia.org/wiki/Dorsalgia) |
| S32 | [Fracture](http://wikipedia.org/wiki/Fracture) of [lumbar spine](http://wikipedia.org/wiki/Lumbar_spine) and [pelvis](http://wikipedia.org/wiki/Human_pelvis) |
| S22 | [Fracture of rib](http://wikipedia.org/wiki/Rib_fracture)(s), [sternum](http://wikipedia.org/wiki/Human_sternum) and [thoracic spine](http://wikipedia.org/wiki/Thoracic_spine) |
| S12 | [Fracture of neck](http://wikipedia.org/wiki/Fracture_of_neck) |
| **Exclusion codes** | |
| A00-B99 | Certain infectious and parasitic diseases |
| C00-D48 | Neoplasms |
| M490 | [Tuberculosis](http://wikipedia.org/wiki/Tuberculosis) of the [spine](http://wikipedia.org/wiki/Vertebral_column) |
| M491 | Brucella spondylitis |
| M492 | Enterobacterial spondylitis |
| M493 | [Spondylopathy](http://wikipedia.org/wiki/Spondylopathy) in other [infectious](http://wikipedia.org/wiki/Infection) and [parasitic diseases](http://wikipedia.org/wiki/Parasitic_diseases) classified elsewhere |

KCD, Korean Standard Classification of Disease.

**Supplementary Table S2.** Procedure codes according to the type and complexity of surgery

|  | **Procedure codes** |
| --- | --- |
| **Types of surgery** |  |
| Fusion | N0444, N0445, N0446, N0447,  N2461, N2462, N2463, N2464, N2465, N2466, N0466, N1466, N2467, N2468, N2469, N0468, N0469, N1469, N2470, N1460 |
| Open discectomy | N1493, N1491, N1492 |
| PELD | N1494 |
| Nucleolysis | N1495, N1496 |
| Pt_plasty | N0471, N0472, N0473, N0474, N0475 |
| Laminectomy | 1499, N2499, N1497, N2497, N1498, N2498 |
| C_plasty | N2491, N2492 |
| Corpectomy | N0451, N0452, N0453 |
| Reduction | N0630, N0591 |
| Others | N0480, N2471, N2472 |
| **Complexity of surgery** |  |
| Percutaneous | N0471, N0472, N0473, N0474, N0475, N1494, N1495, N1496, N0630 |
| Open (simple) | N2491, N2492, N1491, N1492, N1493,  N1499, N1497, N1498, N2499, N2497, N2498,  N0451, N0452, N0453, N0591,  N0454, N0455, N2471, N2472 |
| Open (with instrument) | N0444, N0445, N0446, N0447,  N2461, N2462, N2463, N2464, N2465, N2466, N0466, N1466, N2467, N2468, N2469, N0468, N0469, N1469, N2470, N1460 |

PELD, percutaneous endoscopic lumbar decompression; Pt_plasty, percutaneous plasty; C_plasty, cervical spine laminoplasty.

**Supplementary Table S3.** KCD-7 according to disease classification

| **Classification** | **Codes** |
| --- | --- |
| **Types of disease** |  |
| Deforming dorsopathies | M40, M41, M41, M42, M43 |
| Inflammatory spondylopathies | M45, M46 |
| Stenosis | M480 |
| Spondylosis | M47 |
| Other spondylopathies | M48(M480제외), M494, M495, M498 |
| Cervical disc disorders | M50 |
| Lumbar disc disorders | M51 |
| Dorsopathy | M53 |
| Dorsalgia | M54 |
| Fracture | S12, S22, S32 |
| **Disease sites** |  |
| C-spine | M4001, M4002, M4003, M4011, M4012, M4013, M4021, M4022, M4023, M4031, M4032, M4033, M4041, M4042, M4043, M4051, M4052, M4053, M4101, M4102, M4103,  M4111, M4112, M4113, M4121, M4122, M4123, M4131, M4132, M4133, M4141, M4142, M4143, M4151, M4152, M4153, M4161, M4162, M4163, M4171, M4172, M4173, M4181, M4182, M4183, M4191, M4192, M4193, M4201, M4202, M4203, M4211, M4212, M4213, M4291, M4292, M4293, M4301, M4302, M4303, M4311, M4312, M4313,  M4321, M4322, M4323, M4351, M4352, M4353, M4381, M4382, M4383, M4391, M4392, M4393, M4601, M4602, M4603, M4621, M4622, M4623, M4631, M4632, M4633, M4641, M4642, M4643, M4651, M4652, M4653, M4681, M4682, M4683, M4691, M4692, M4693, M4701, M4702, M4703, M4711, M4712, M4713, M4721, M4722, M4723,  M4781, M4782, M4783, M4791, M4792, M4793, M4801, M4802, M4803, M4811, M4812, M4813, M4821, M4822, M4823, M4831, M4832, M4833, M4841, M4842, M4843, M4851, M4852, M4853, M4881, M4882, M4883, M4891, M4892, M4893, M4941, M4942, M4943, M4951, M4952, M4953, M4981, M4982, M4983, M5321, M5322, M5323,  M5381, M5382, M5383, M5391, M5392, M5393, M5401, M5402, M5403, M5411, M5412, M5413, M5481, M5482, M5483, M5491, M5492, M5493,  M451, M452, M453, M433, M434, M436, M530, M531, M542, M50, S12 |
| T-spine | M4004, M4014, M4024, M4034, M4044, M4054, M4104, M4114, M4124, M4134, M4144, M4154, M4164, M4174, M4184, M4194, M4204, M4214, M4294, M4304, M4314, M4324, M4354, M4384, M4394, M4604, M4624, M4634, M4644, M4654, M4684, M4694, M4704, M4714, M4724,  M4784, M4794, M4804, M4814, M4824, M4834, M4844, M4854, M4884, M4894, M4944, M4954, M4984, M5324, M5384, M5394, M5404, M5414, M5484, M5494,  M454, M546, S22 |
| L-spine | M4005, M4006, M4007, M4008, M4015, M4016, M4017, M4018, M4025, M4026, M4027, M4028, M4035, M4036, M4037, M4038, M4045, M4046, M4047, M4048, M4055, M4056, M4057, M4058, M4105, M4106, M4107, M4108,  M4115, M4116, M4117, M4118, M4125, M4126, M4127, M4128, M4135, M4136, M4137, M4138, M4145, M4146, M4147, M4148, M4155, M4156, M4157, M4158, M4165, M4166, M4167, M4168, M4175, M4176, M4177, M4178, M4185, M4186, M4187, M4188, M4195, M4196, M4197, M4198, M4205, M4206, M4207, M4208, M4215, M4216, M4217, M4218, M4295, M4296, M4297, M4298, M4305, M4306, M4307, M4308, M4315, M4316, M4317, M4318,  M4325, M4326, M4327, M4328, M4355, M4356, M4357, M4358, M4385, M4386, M4387, M4388, M4395, M4396, M4397, M4398, M4605, M4606, M4607, M4608, M4625, M4626, M4627, M4628, M4635, M4636, M4637, M4638, M4645, M4646, M4647, M4648, M4655, M4656, M4657, M4658, M4685, M4686, M4687, M4688, M4695, M4696, M4697, M4698, M4705, M4706, M4707, M4708, M4715, M4716, M4717, M4718, M4725, M4726, M4727, M4728,  M4785, M4786, M4787, M4788, M4795, M4796, M4797, M4798, M4805, M4806, M4807, M4808, M4815, M4816, M4817, M4818, M4825, M4826, M4827, M4828, M4835, M4836, M4837, M4838, M4845, M4846, M4847, M4848, M4855, M4856, M4857, M4858, M4885, M4886, M4887, M4888, M4895, M4896, M4897, M4898, M4945, M4946, M4947, M4948, M4955, M4956, M4957, M4958, M4985, M4986, M4987, M4988, M5325, M5326, M5327, M5328,  M5385, M5386, M5387, M5388, M5395, M5396, M5397, M5398, M5405, M5406, M5407, M5408, M5415, M5416, M5417, M5418, M5485, M5486, M5487, M5488, M5495, M5496, M5497, M5498,  M455, M456, M457, M458, M461, M533, M543, M544, M545, M51, S32 |
| Others | M4000, M4009, M4010, M4019, M4020, M4029, M4030, M4039, M4040, M4049, M4050, M4059, M4100, M4109, M4110, M4119, M4120, M4129, M4130, M4139, M4140, M4149, M4150, M4159, M4160, M4169, M4170, M4179,  M4180, M4189, M4190, M4199, M4200, M4209, M4210, M4219, M4290, M4299, M4300, M4309, M4310, M4319, M4320, M4329, M4350, M4359, M4380, M4389, M4390, M4399, M4600, M4609, M4620, M4629, M4630, M4639, M4640, M4649, M4650, M4659, M4680, M4689, M4690, M4699, M4700, M4709, M4710, M4719, M4720, M4729, M4780, M4789, M4790, M4799, M4800, M4809, M4810, M4819, M4820, M4829, M4830, M4839, M4840, M4849,  M4850, M4859, M4880, M4889, M4890, M4899, M4940, M4949, M4950, M4959, M4980, M4989, M5320, M5329, M5380, M5389, M5390, M5399, M5400, M5409, M5410, M5419, M5480, M5489, M5490, M5499,  M450, M459 |

KCD, Korean Standard Classification of Disease.

**Supplementary Table S4.** KM expenditure and the time to the first use of KM (mean)

|  | **KM expenditure** | | **Time to the first use of KM** | |
| --- | --- | --- | --- | --- |
|  | **Mean** | ***p*-value** | **Mean** | ***p*-value** |
| **Total (N = 5711)** | 492.11 ± 918.64 |  | 522.05±490.23 |  |
| **Sex** |  |  |  |  |
| Male | 407.7 ± 825.69 | <.0001 | 545.55 ± 503.59 | 0.0036 |
| Female | 510.94 ± 916.41 |  | 506.85 ± 480.85 |  |
| **Age (years)** |  |  |  |  |
| 0-19 | 258.04 ± 381.01 | <.0001 | 646.2 ± 519.67 | <.0001 |
| 20-29 | 214.21 ± 558.37 |  | 711.61 ± 506.76 |  |
| 30-39 | 331.36 ± 744.06 |  | 650.29 ± 538.3 |  |
| 40-49 | 454.81 ± 1200.22 |  | 539.48 ± 504.53 |  |
| 50-59 | 444.07 ± 957.07 |  | 549.75 ± 507.31 |  |
| 60-69 | 503.93 ± 831.71 |  | 535.42 ± 485.84 |  |
| ≥70 | 517.83 ± 789.6 |  | 449.11 ± 453.45 |  |
| **Income** |  |  |  |  |
| Level 1 (low) | 500.27 ± 924.02 | 0.4042 | 513.77 ± 478.84 | 0.3021 |
| Level 2 | 472.96 ± 990.27 |  | 541.25 ± 500.32 |  |
| Level 3 | 477.09 ± 934.36 |  | 527.12 ± 492.66 |  |
| Level 4 | 445.12 ± 715.07 |  | 509.62 ± 489.38 |  |
| **Region** |  |  |  |  |
| Seoul | 517.84 ± 1076.63 | 0.0003 | 544.29 ± 495.09 | <.0001 |
| Capital area | 414.49 ± 725.35 |  | 565.91 ± 505.25 |  |
| Metropolitan city | 552.22 ± 1041.95 |  | 499.45 ± 482.7 |  |
| Others | 450.43 ± 810.7 |  | 496.15 ± 479.89 |  |
| **CCI^3^** |  |  |  |  |
| 0 | 391.07 ± 922.98 | <.0001 | 599.86 ± 524.7 | <.0001 |
| 1 | 408.57 ± 832.64 |  | 545.56 ± 502.71 |  |
| 2 | 451.21 ± 807.81 |  | 526.91 ± 481.71 |  |
| 3 | 508.94 ± 810.59 |  | 469.98 ± 473.68 |  |
| ≥4 | 576.65 ± 993.27 |  | 475.04 ± 462.4 |  |
| **Use of KM before surgery** |  |  |  |  |
| None | 379.43 ± 793.47 | <.0001 | 625.48 ± 504.77 | <.0001 |
| KM use | 540.44 ± 940.76 |  | 442.39 ± 463.33 |  |
| **Types of surgery** |  |  |  |  |
| Fusion | 509.35 ± 1026.63 | 0.0431 | 541.53 ± 480.67 | 0.0686 |
| Open discectomy | 456.35 ± 943.49 | 0.1544 | 545.39 ± 492.1 | <.0001 |
| PELD | 339.93 ± 508.8 | 0.2311 | 528.97 ± 474.05 | 0.909 |
| Nucleolysis | 473.86 ± 429.49 | 0.9891 | 453.17 ± 371.98 | 0.6261 |
| Pt_plasty | 520.12 ± 816.08 | 0.0311 | 444.85 ± 468.7 | <.0001 |
| Laminectomy | 498.84 ± 772.64 | 0.2055 | 525.87 ± 496.07 | 0.7597 |
| C_plasty | 502.85 ± 914.28 | 0.8023 | 482.02 ± 510.81 | 0.5782 |
| Corpectomy | 649.41 ± 950.34 | 0.3639 | 502.7 ± 501.56 | 0.8596 |
| Reduction | 146.75 ± 137.99 | 0.1554 | 304.47 ± 314.77 | 0.0852 |
| Others | 524.03 ± 610.72 | 0.7753 | 408.27 ± 423.99 | 0.2754 |
| **Complexity of surgery** |  |  |  |  |
| Percutaneous | 501.87 ± 799.2 | 0.013 | 446.77 ± 466.75 | <.0001 |
| Open (simple) | 437.32 ± 833.21 |  | 542.72 ± 501.54 |  |
| Open (w. instrument) | 509.35 ± 1026.63 |  | 541.53 ± 480.67 |  |
| **Types of disease** |  |  |  |  |
| Lumbar disc disorders | 425.88 ± 844.89 | 0.0784 | 545.89 ± 497.91 | <.0001 |
| Fracture | 495.04 ± 787.03 |  | 450.52 ± 467.26 |  |
| Stenosis | 510.36 ± 858.97 |  | 530.74 ± 489.95 |  |
| Deforming dorsopathies | 556.38 ± 1266.46 |  | 545.13 ± 489.46 |  |
| Cervical disc disorders | 425.48 ± 902.22 |  | 567.26 ± 513.81 |  |
| Spondylosis | 392.67 ± 798.33 |  | 507.17 ± 473.48 |  |
| Inflammatory spondylopathies | 332.75 ± 340.47 |  | 731.73 ± 406.35 |  |
| Dorsalgia | 538.85 ± 920.08 |  | 458.24 ± 494.47 |  |
| Dorsopathy | 454.15 ± 1160.29 |  | 673.2 ± 447.69 |  |
| Other spondylopathies | 481.81 ± 913.85 |  | 354.7 ± 391.6 |  |
| **Surgical sites** |  |  |  |  |
| C_spine | 426.43 ± 891.67 | 0.5589 | 566.85 ± 512.46 | 0.0008 |
| T_spine | 506.91 ± 826.07 |  | 448.58 ± 467.31 |  |
| L_spine | 470.65 ± 887.49 |  | 526.44 ± 489.83 |  |
| Others | 480.53 ± 945.22 |  | 473.94 ± 480.79 |  |
| **Type of institutions** |  |  |  |  |
| Clinic | 550,394 ± 861,235 | 0.821 | 472 ± 501.83 | 0.3874 |
| Hospital | 532,957 ± 1,039,321 |  | 526.37 ± 494.85 |  |
| General hospital | 504,584 ± 800,660 |  | 510.62 ± 489.12 |  |
| Tertiary hospital | 530,708 ± 1,078,089 |  | 533.37 ± 467.66 |  |

KM, Korean medicine; PELD, percutaneous endoscopic lumbar decompression; Pt_plasty, percutaneous plasty; C_plasty, cervical spine laminoplasty.

**Supplementary table S5.** KM treatments after spin surgery

|  | **Claims** | **Expenditure** |
| --- | --- | --- |
|  | **N (%)** | **US dollars (%)** |
| **Total** | 119,925 | 2,505,645 |
| **Acupuncture** |  |  |
| Basic acupuncture | 112,744 (94.0) | 443,654 (17.7) |
| Special acupuncture |  |  |
| Penetration needling | 44,098 (36.8) | 228,498 (9.1) |
| Intra-articular | 38,287 (31.9) | 101,552 (4.1) |
| Intervertebral | 22,956 (19.1) | 62,643 (2.5) |
| Intra-abdominal | 1,056 (0.9) | 2,851 (0.1) |
| Electro | 135 (0.1) | 1,248 (0.0) |
| Laser | 112 (0.1) | 297 (0.0) |
| Intra-nasal | 77 (0.1) | 146 (0.0) |
| Intra-orbital | 39 (0.0) | 66 (0.0) |
| Others | 168 (0.1) | 660 (0.0) |
| **Electroacupuncture** | 26,809 (22.4) | 112,923 (4.5) |
| **Chuna manual therapy** |  |  |
| Complex | 22 (0.0) | 618 (0.0) |
| Simple | 33 (0.0) | 549 (0.0) |
| **Moxibustion** | 28,273 (23.6) | 93,960 (3.7) |
| **Cupping** |  |  |
| Wet cupping | 17,923 (14.9) | 139,425 (5.6) |
| Dry cupping | 34,446 (28.7) | 106,183 (4.2) |
| **Warm meridian** | 50,149 (41.8) | 39,964 (1.6) |
| **Tests** |  |  |
| Meridian function test | 244 (0.2) | 820 (0.0) |
| Electro-pulse graph | 29 (0.0) | 71 (0.0) |
| Yangdorak test | 16 (0.0) | 42 (0.0) |

**Supplementary Table S6.** Factors affecting the use of KM at 3 years after spinal surgery

|  | **Probability (logit)** | **Conditional (GLM)** | | **Marginal effects** |
| --- | --- | --- | --- | --- |
|  | **OR (95% CI)** | **Coeff (SE)** | ***p*-value** | **Margin (95% CI)** |
| **Sex (ref = male)** |  |  |  |  |
| Male |  |  |  | 126.89 (115.22 to 138.57) |
| Female | 1.36 (1.25 to 1.48) | 0.09 (0.06) | 0.091 | 162.85 (151.28 to 174.43) |
| **Age (ref = <30)** |  |  |  |  |
| <30 |  |  |  | 125.41 (107.40 to 143.42) |
| 30-50 | 1.23 (1.09 to 1.39) | 0.03 (0.09) | 0.691 | 145.36 (126.65 to 164.08) |
| 50-70 | 1.54 (1.35 to 1.75) | -0.05 (0.09) | 0.580 | 149.89 (133.07 to 166.70) |
| ≥70 | 1.63 (1.42 to 1.86) | 0.01 (0.09) | 0.953 | 162.26 (145.94 to 178.59) |
| **Region (ref = Seoul)** |  |  |  |  |
| Seoul |  |  |  | 165.17 (140.67 to 189.66) |
| Capital area | 1.01 (0.89 to 1.15) | -0.28 (0.09) | 0.001 | 125.24 (111.46 to 139.01) |
| Metropolitan city | 1.31 (1.14 to 1.50) | -0.05 (0.09) | 0.604 | 180.08 (157.48 to 202.67) |
| Others | 1.26 (1.12 to 1.42) | -0.27 (0.08) | 0.001 | 141.80 (129.70 to 153.91) |
| **CCI (ref = 0)** |  |  |  |  |
| 0 |  |  |  | 115.20 (98.37 to 132.03) |
| 1 | 1.26 (1.12 to 1.43) | 0.04 (0.09) | 0.641 | 135.87 (120.11 to 151.64) |
| 2 | 1.31 (1.15 to 1.48) | 0.05 (0.09) | 0.552 | 140.03 (123.26 to 156.79) |
| 3 | 1.57 (1.36 to 1.82) | 0.14 (0.10) | 0.150 | 166.94 (144.14 to 189.74) |
| ≥ 4 | 1.47 (1.29 to 1.67) | 0.24 (0.09) | 0.006 | 179.19 (159.14 to 199.24) |
| **Use of KM before surgery (ref = none)** |  |  |  |  |
| None |  |  |  | 91.21 (83.14 to 99.29) |
| KM use | 2.93 (2.70 to 3.17) | 0.29 (0.05) | 0.000 | 218.86 (203.26 to 234.46) |
| **Type of surgery (ref = decom)** |  |  |  |  |
| Decom (n = 4724) |  |  |  | 132.91 (115.40 to 150.42) |
| Fusion (n = 3025) | 0.94 (0.79 to 1.10) | 0.05 (0.11) | 0.640 | 135.03 (114.63 to 155.43) |
| Pt_plasty (n = 2340) | 0.89 (0.67 to 1.18) | 0.53 (0.19) | 0.005 | 212.40 (144.12 to 280.69) |
| Others (n = 1278) | 1.04 (0.88 to 1.22) | 0.10 (0.10) | 0.348 | 148.88 (122.64 to 175.12) |
| **Types of disease (ref = lumbar disc disorders)** |  |  |  |  |
| Lumbar disc disorders |  |  |  | 170.19 (141.30 to 199.07) |
| Fracture | 0.87 (0.67 to 1.14) | -0.42 (0.18) | 0.022 | 104.33 (77.84 to 130.82) |
| Stenosis | 1.02 (0.89 to 1.18) | -0.01 (0.09) | 0.912 | 170.48 (142.81 to 198.16) |
| Deforming dorsopathies | 0.87 (0.72 to 1.06) | 0.08 (0.13) | 0.533 | 172.05 (130.98 to 213.12) |
| Cervical disc disorders | 1.15 (0.94 to 1.40) | -0.19 (0.13) | 0.162 | 150.74 (112.18 to 189.30) |
| Others | 1.00 (0.77 to 1.29) | -0.22 (0.16) | 0.192 | 137.06 (94.83 to 179.29) |
| **Costs of surgery (ref = Q1)** |  |  |  |  |
| Q1 |  |  |  | 150.65 (127.59 to 173.70) |
| Q2 | 0.98 (0.86 to 1.11) | -0.11 (0.09) | 0.200 | 133.35 (116.56 to 150.14) |
| Q3 | 1.05 (0.91 to 1.21) | -0.01 (0.10) | 0.879 | 152.13 (134.86 to 169.39) |
| Q4 | 1.04 (0.88 to 1.24) | 0.00 (0.12) | 0.981 | 154.19 (131.71 to 176.66) |
| **Length of stay (ref = 0)** |  |  |  |  |
| Q1 |  |  |  | 133.39 (114.70 to 152.08) |
| Q2 | 1.00 (0.88 to 1.14) | 0.01 (0.08) | 0.922 | 134.73 (118.39 to 151.07) |
| Q3 | 1.03 (0.90 to 1.18) | 0.10 (0.09) | 0.278 | 149.75 (133.22 to 166.29) |
| Q4 | 1.05 (0.90 to 1.22) | 0.21 (0.10) | 0.033 | 168.74 (148.16 to 189.31) |

KM, Korean medicine; GLM, generalized linear model; Decom, decompression surgery; Pt_plasty, percutaneous plasty; OR, odds ratio; SE, standard error; CI, confidence interval; CCI, Charlson Comorbidity Index.

**Supplementary Table S7.** Factors affecting the use of KM at 1 year after spinal surgery

|  | **Probability (logit)** | **Conditional (GLM)** | | **Marginal effects** |
| --- | --- | --- | --- | --- |
|  | **OR (95% CI)** | **Coeff (SE)** | ***p*-value** | **Margin (95% CI)** |
| **Sex (ref = male)** |  |  |  |  |
| Male |  |  |  | 52.42 (46.37 to 58.46) |
| Female | 1.22 (1.11 to 1.35) | -0.05 (0.07) | 0.423 | 57.12 (52.14 to 62.10) |
| **Age (ref = <30)** |  |  |  |  |
| <30 |  |  |  | 48.94 (40.22 to 57.65) |
| 30-50 | 1.26 (1.09 to 1.45) | 0.00 (0.10) | 0.970 | 57.84 (48.66 to 67.02) |
| 50-70 | 1.35 (1.16 to 1.57) | -0.16 (0.10) | 0.127 | 51.73 (44.44 to 59.01) |
| ≥70 | 1.50 (1.29 to 1.75) | -0.09 (0.10) | 0.406 | 59.65 (52.42 to 66.89) |
| **Region (ref = Seoul)** |  |  |  |  |
| Seoul |  |  |  | 60.61 (49.42 to 71.81) |
| Capital area | 1.00 (0.86 to 1.15) | -0.25 (0.10) | 0.017 | 47.32 (40.78 to 53.87) |
| Metropolitan city | 1.29 (1.10 to 1.51) | -0.08 (0.11) | 0.455 | 66.60 (56.55 to 76.64) |
| Others | 1.23 (1.07 to 1.41) | -0.27 (0.10) | 0.005 | 52.96 (47.41 to 58.52) |
| **CCI (ref = 0)** |  |  |  |  |
| 0 |  |  |  | 43.22 (35.38 to 51.06) |
| 1 | 1.22 (1.06 to 1.41) | 0.02 (0.10) | 0.866 | 50.75 (43.38 to 58.12) |
| 2 | 1.27 (1.10 to 1.48) | 0.04 (0.11) | 0.685 | 53.64 (45.66 to 61.62) |
| 3 | 1.58 (1.35 to 1.87) | -0.02 (0.11) | 0.880 | 58.51 (48.93 to 68.10) |
| ≥ 4 | 1.47 (1.26 to 1.71) | 0.18 (0.10) | 0.089 | 67.42 (58.33 to 76.50) |
| **Use of KM before surgery (ref = none)** |  |  |  |  |
| None |  |  |  | 34.02 (30.01 to 38.02) |
| KM use | 3.28 (2.99 to 3.59) | 0.02 (0.06) | 0.804 | 82.03 (75.14 to 88.91) |
| **Type of surgery (ref = decom)** |  |  |  |  |
| Decom (n = 4724) |  |  |  | 44.62 (37.37 to 51.87) |
| Fusion (n = 3025) | 0.96 (0.79 to 1.15) | 0.13 (0.12) | 0.289 | 49.29 (39.92 to 58.65) |
| Pt_plasty (n = 2340) | 1.27 (0.91 to 1.76) | 0.56 (0.23) | 0.017 | 91.39 (53.85 to 128.94) |
| Others (n = 1278) | 1.24 (1.04 to 1.49) | 0.20 (0.12) | 0.093 | 63.22 (49.81 to 76.62) |
| **Types of disease (ref = lumbar disc disorders)** |  |  |  |  |
| Lumbar disc disorders |  |  |  | 64.95 (50.57 to 79.34) |
| Fracture | 0.86 (0.63 to 1.18) | -0.39 (0.23) | 0.086 | 39.64 (27.00 to 52.29) |
| Stenosis | 0.97 (0.83 to 1.15) | -0.01 (0.11) | 0.935 | 63.22 (50.03 to 76.40) |
| Deforming dorsopathies | 0.86 (0.68 to 1.07) | 0.06 (0.16) | 0.723 | 61.72 (42.50 to 80.94) |
| Cervical disc disorders | 1.08 (0.86 to 1.36) | -0.28 (0.16) | 0.090 | 51.93 (34.73 to 69.12) |
| Others | 0.96 (0.72 to 1.28) | -0.08 (0.20) | 0.686 | 58.34 (36.08 to 80.60) |
| **Costs of surgery (ref = Q1)** |  |  |  |  |
| Q1 |  |  |  | 53.27 (43.33 to 63.20) |
| Q2 | 1.01 (0.87 to 1.17) | -0.08 (0.10) | 0.442 | 49.56 (41.92 to 57.21) |
| Q3 | 1.14 (0.97 to 1.34) | 0.04 (0.11) | 0.741 | 60.42 (52.22 to 68.62) |
| Q4 | 1.00 (0.82 to 1.23) | 0.05 (0.13) | 0.701 | 56.24 (46.24 to 66.24) |
| **Length of stay (ref = 0)** |  |  |  |  |
| Q1 |  |  |  | 48.37 (39.99 to 56.76) |
| Q2 | 1.01 (0.88 to 1.17) | -0.01 (0.10) | 0.948 | 48.49 (41.17 to 55.81) |
| Q3 | 1.12 (0.96 to 1.31) | 0.04 (0.11) | 0.695 | 54.52 (47.29 to 61.75) |
| Q4 | 1.05 (0.88 to 1.24) | 0.29 (0.12) | 0.013 | 66.70 (56.61 to 76.78) |

KM, Korean medicine; GLM, generalized linear model; Decom, decompression surgery; Pt_plasty, percutaneous plasty; OR, odds ratio; SE, standard error; CI, confidence interval; CCI, Charlson Comorbidity Index.

0
